# Determinants of growth parameters at 12-month corrected age among very preterm infants in China: a retrospective cohort study

**DOI:** 10.3389/fped.2026.1847139

**Published:** 2026-07-06

**Authors:** Wenting Zheng, Youjiong Wang, Jinfeng Liu, Lei Ye, Yuanyuan Zhang, Jie Cao, Lijin Zhao

**Affiliations:** 1 Department of Neonatology and Obstetric Ward, Shanghai First Maternity and Infant Hospital, School of Medicine, Tongji University, Shanghai, China; 2 Department of Neonatology, First Peoples of Hospital of Taicang, Taicang, China; 3 Tongji University School of Medicine, Shanghai, China

**Keywords:** corrected age, growth determinants, maternal postnatal depression, physical growth, very preterm infant

## Abstract

**Background:**

Preterm birth is a leading cause of infant mortality and morbidity, with very preterm infants (VPI) being particularly vulnerable to poor physical growth and developmental challenges. This study aimed to investigate the trends and factors influencing physical growth in VPI within the first 12 months of corrected age (CA).

**Methods:**

A cohort study was conducted on 223 VPI admitted between November 2020 and July 2022 at a tertiary maternity and neonatal hospital in Shanghai, China. Multiple linear regression and logistic regression models were used to identify risk factors for weight, length, and head circumference growth outcomes within 12 months CA.

**Results:**

The results showed that maternal postnatal depression (*p* < 0.05), the presence of clinical hypothyroidism, gestational diabetes mellitus (GDM), preeclampsia in mothers and VPI with congenital heart diseases were risk factors for physical growth. Girls with greater gestational age and birth weight, a higher 1-minute Apgar score, and exclusive breastfeeding have better physical growth outcomes.

**Conclusion:**

Targeted interventions addressing maternal mental health, pregnancy complications, and infant-specific risk factors are warranted to promote physical growth in VPI.

## 1 Introduction

Preterm birth (PTB), defined as delivery before 37 completed weeks of gestation, is recognized by the World Health Organization as the leading cause of neonatal mortality and morbidity globally (WHO, 2009) (1). Preterm infants face elevated risks of short- and long-term health complications, imposing substantial emotional and economic burdens on families and public health systems. Despite extensive research, the global incidence of PTB has not declined, and its multifactorial etiology remains incompletely understood (2). Developmental impairments in preterm infants remain underrecognized in public health policy and clinical practice.

Infants born between 28 and 32 weeks of gestation are classified as **very preterm infants (VPI)**. Accounting for approximately 11.3% of all preterm births, VPI are highly vulnerable due to immature organ function, weakened immunity, and prolonged neonatal intensive care unit (NICU) stays. Even after successful discharge, VPI experience high rates of post-discharge growth failure (3). Previous studies indicate that 70%–90% of VPI exhibit growth parameters below the 50th percentile, and 25%–50% fall below the 10th percentile of reference standards (4). Early-life growth restriction and nutritional deficits are linked to impaired brain development, suboptimal neurocognitive outcomes, and increased susceptibility to chronic non-communicable diseases in adulthood, including hypertension, type 2 diabetes, obesity, and hypercholesterolemia (5).

Perinatal factors have been attached with more importance to the growth outcome of preterm infants from birth to 12 months corrected age (CA). However, high-quality population-specific data on VPI growth patterns and modifiable risk factors remain limited, especially in mainland China. To address this gap, the present study analyzed perinatal and postnatal factors associated with physical growth in VPI from 1 to 12 months CA. The findings aim to provide an evidence base for individualized post-discharge growth monitoring and developmental support for VPI.

## 2 Aims

This study characterized longitudinal growth trajectories (weight, length, and head circumference) in VPI from 1 to 12 months CA. We further examined associations between maternal characteristics, NICU-related factors, postnatal care practices, and growth outcomes across this period. Improved understanding of adverse growth determinants can enable pediatric clinicians to identify high-risk infants and implement targeted monitoring and interventions to prevent growth faltering.

## 3 Methods

### 3.1 Study design and participants

This prospective cohort study with a 12-month follow-up period was conducted from November 2020 to July 2022 at a tertiary maternity and neonatal hospital in Shanghai, China. Mother-infant dyads were enrolled by convenience sampling during outpatient follow-up clinics.

Inclusion Criteria:

1. Infants were born at 28–32 weeks of gestation (very preterm infants).
2. Infants survived to 12 months corrected age (CA) and completed all follow-up assessments.
3. Mothers completed the postpartum depression questionnaire during the infant's hospitalization.
4. Complete baseline and follow-up anthropometric and clinical data were available for analysis.

Exclusion Criteria

1. Infants with congenital malformations or genetic syndromes that may affect physical growth and development.
2. Infants with surgical diseases, especially necrotizing enterocolitis (NEC) requiring surgical intervention.
3. Infants with severe postnatal complications leading to transfer to other hospitals or loss to follow-up.
4. Infants who died during hospitalization or the follow-up period.
5. Mothers with severe pre-existing psychiatric disorders or incomplete questionnaire data.

A total of 223 mother-infant dyads that met all inclusion criteria and none of the exclusion criteria were finally included in the analysis.

### 3.2 Data collection

#### 3.2.1 Sample size calculation

The sample size was calculated using PASS 15.0 software based on a previous study of growth outcomes in very preterm infants. The primary outcome was the z-score of physical growth at 12 months CA.

Assuming a significance level (*α*) of 0.05 (two-sided), a test power (1−*β*) of 0.80, and an expected effect size of 0.30 for the association between maternal postnatal depression and infant growth, the minimum required sample size was 200 infants.

Considering an anticipated 10% loss to follow-up (transfer to other hospitals, death, or incomplete data), the target sample size was expanded to 220 infants.

The final analytic sample of 223 very preterm infants was consistent with this calculation and provided sufficient statistical power for the regression analyses.

#### 3.2.2 Data collection procedures

Anthropometric measurements (weight, length, head circumference) were obtained at nine time points: 1 (T1), 2 (T2), 3 (T3), 4 (T4), 5 (T5), 6 (T6), 8 (T7), 10 (T8), and 12 (T9) months CA. Initially, 279 VPI (28–32 weeks) were assessed. Forty-seven infants were excluded due to transfer to other hospitals and loss to follow-up, and nine infants died during hospitalization. The final analytic sample comprised 223 mother–infant dyads. Follow-up attendance varied slightly across time points due to infant illness or parental scheduling constraints: 211, 213, 201, 212, 199, 208, 214, 206, and 216 infants at T1–T9, respectively. Maternal postpartum depression was assessed during the infant's hospitalization in the neonatal intensive care unit (NICU). All data were collected and double-checked by two trained researchers (Zhao Lijin and Cao Jie) to ensure accuracy and consistency.

### 3.3 Measures

#### 3.3.1 Baseline characteristics

Maternal data included age, educational level, mode of delivery, and pregnancy complications [preterm premature rupture of membranes [PROM], cervical insufficiency, preeclampsia, gestational diabetes mellitus [GDM], subclinical hypothyroidism, and pregnancy-induced hypertension] extracted from the hospital information system (HIS).

Infant data included gestational age, birth weight, sex, 1-minute and 5-minute Apgar scores, multiple birth status, congenital heart disease (CHD), anemia of prematurity, bronchopulmonary dysplasia (BPD), NICU length of stay, and in-hospital feeding modality (exclusive breastfeeding, mixed feeding, exclusive formula feeding). Neonatal morbidities including acute respiratory distress syndrome (NRDS), hypoglycemia, hyperbilirubinemia, and CHD were abstracted from admission and discharge records.

#### 3.3.2 Physical growth assessment

Weight, length, and head circumference were measured using calibrated instruments in a dedicated clinic room by trained staff. Infants were assessed supine, unclothed, and without shoes. Measurements were recorded to the nearest 10 g (weight), 1 cm (length), and 0.1 cm (head circumference). All data were entered into the HIS. Corrected age refers to the time after term time.Growth metrics were converted to age- and sex- standardized z-scores based on the WHO Child Growth Standards (6), which are widely adopted in over 140 countries for evaluating growth from birth to 5 years. The WHO Child Growth Standards provide standard deviation scores (SDS) for length, weight, and head circumference. Therefore, we converted length, weight, and head circumference from 1 to 12 months corrected age (CA) into standardized z scores by gender and age.

#### 3.3.3 Maternal postpartum depression

Maternal postnatal depression (PPD) was assessed using the Edinburgh Postnatal Depression Scale (EPDS), a 10-item self-report tool developed by Cox et al. (7). The Chinese versionvalidated by Lee et al. (8) and revised by Wang Yuqiong et al. (9) was used, showing good cultural adaptation and psychometric properties for Chinese women.

The EPDS quantifies depressive symptoms over the preceding week, with items scored 0–3 and a total score ranging from 0 to 30; higher scores indicate more severe symptoms. A cutoff score of ≥10 was used to define clinically significant PPD in this study. The Chinese EPDS demonstrates sensitivity of 82% and specificity of 86%, with a Cronbach's *α* of 0.85, indicating excellent reliability (10).

In this study, PPD was evaluated at 1 month postpartum. This time point was selected because the prevalence of postpartum depression peaks within 2–6 weeks after delivery, and the first month postpartum represents the high-risk window for clinically significant depressive symptoms. Furthermore, maternal mental health at 1 month postpartum strongly predicts subsequent mother–infant interaction, feeding practices, and long-term infant growth outcomes. All assessments were completed during the infant's hospitalization in the neonatal intensive care unit (NICU) to ensure high compliance and data completeness.

### 3.4 Statistical analysis

Continuous variables were summarized as mean ± standard deviation (SD) or median [interquartile range (IQR)]; categorical variables were presented as counts and percentages (%).

Multiple linear regression was performed to identify predictors of growth z-scores, including maternal age, PPD, GDM, preeclampsia, subclinical hypothyroidism, gestational age, infant sex, birth weight, 1 min Apgar score, multiple birth status, CHD, and in-hospital feeding modality. Preliminary correlation analyses excluded maternal education, gravidity, parity, mode of delivery, NRDS, and NICU length of stay from final models due to non-significant associations with growth outcomes.

Logistic regression was used to analyze factors associated with growth velocity (change in z-scores from 1 to 12 months CA). A change in z-score >0.67 indicated catch-up growth, while a change <0.67 indicated catch-down growth. All statistical tests were two-sided, with *P* < 0.05 considered significant.

## 4 Results

### 4.1 Baseline demographic and clinical characteristics

The final sample included 223 VPI-mother dyads. Baseline characteristics are shown in Table 1. Gestational age ranged from 28 to 32 weeks: 112 (50.2%) infants were born at 28–30 weeks, and 111 (49.8%) at 30–32 weeks.

**Table 1:** General information of the analytic study sample (*n* = 223).

Variables	N (%)	M (±SD)
Information of premature infant		
Male	113 (50.7)	—
Female	110 (49.3)	—
Gestational age	—	30.08 (1.24)
Twins	83（37.2）	
Birth weight (g)	—	1,392.10 (302.48)
1-minute Apgar score	—	7.98 (1.62)
5-minute Apgar score	—	8.87 (1.04)
NRDS	127 (57.2)	—
Congenital heart disease	123 (55.2)	—
Length of stay in NICU	—	55.09 (21.07)
Maternal information		
Maternal age	—	30.99 (3.95)
Mode of delivery: Caesarean	109 (61.9)	—
Gravidity	—	1.78 (1.05)
Parity	—	1.24 (0.46)
Maternal education		
High school and below	35 (15.6)	—
College	34 (15.2)	—
Bachelor degree	105 (47.0)	—
Master degree and above	47 (21.0)	—
Maternal Ppd	91(40.8)	—

M, mean; SD, standard deviation; NRDS, neonatal respiratory distress syndrome; NICU, neonatal intensive care unit.

## 5 Longitudinal growth trajectories in VPI

**Figure 1:**
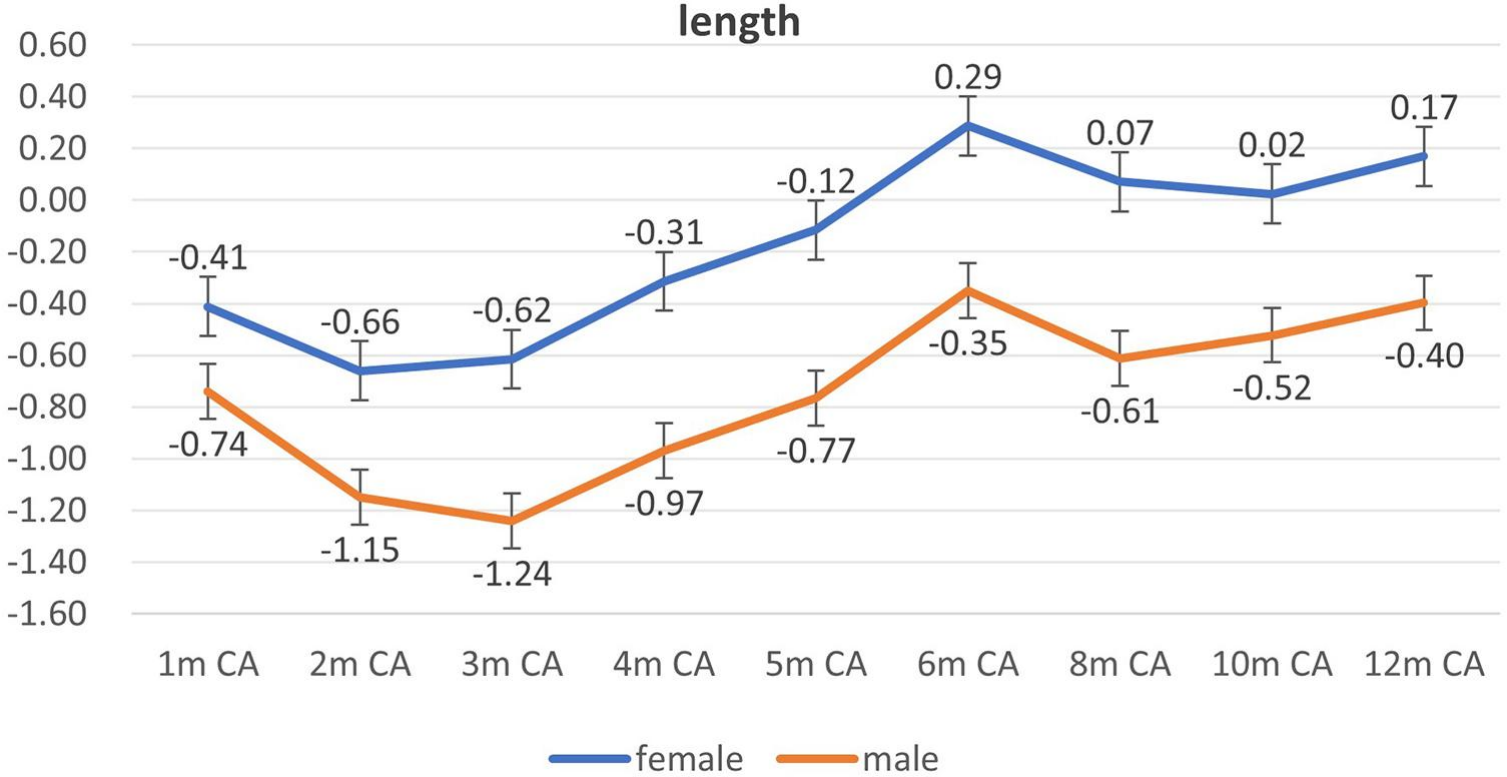
Comparison of Z-scores between sex and length.

**Figure 2:**
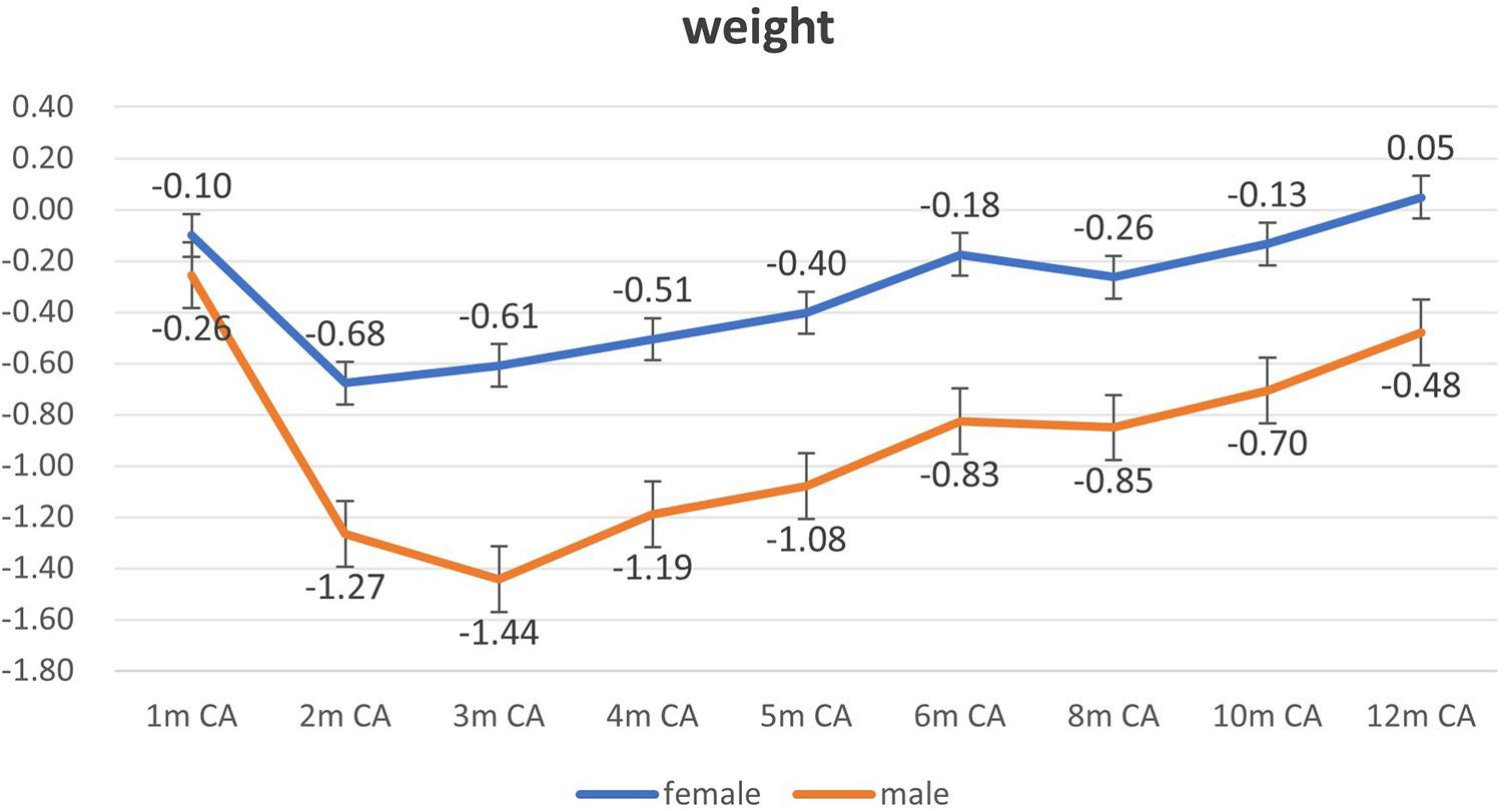
Comparison of Z-scores between sex and weight.

**Figure 3:**
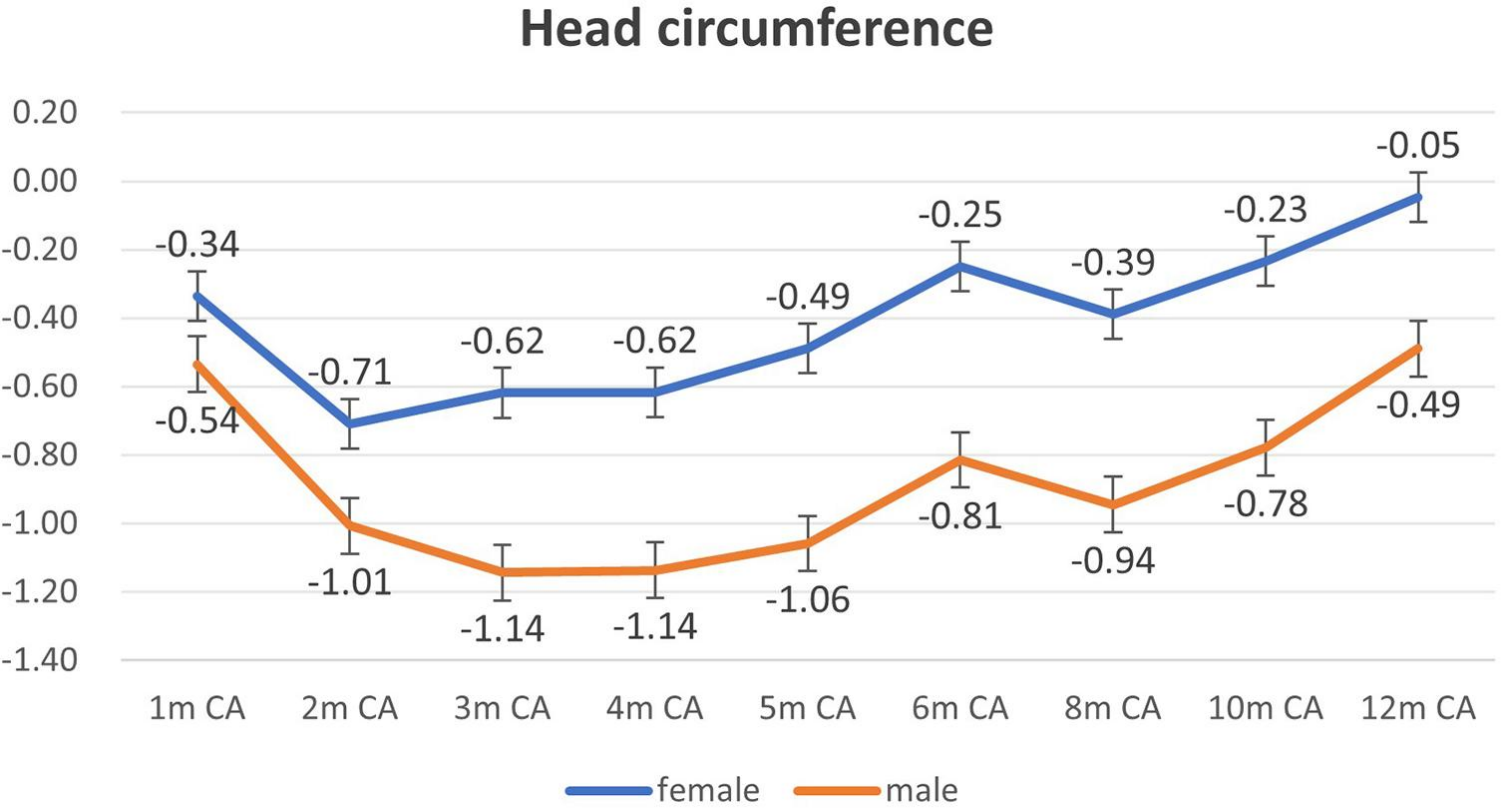
Comparison of Z-scores between sex and head circumference.

**Figure 4:**
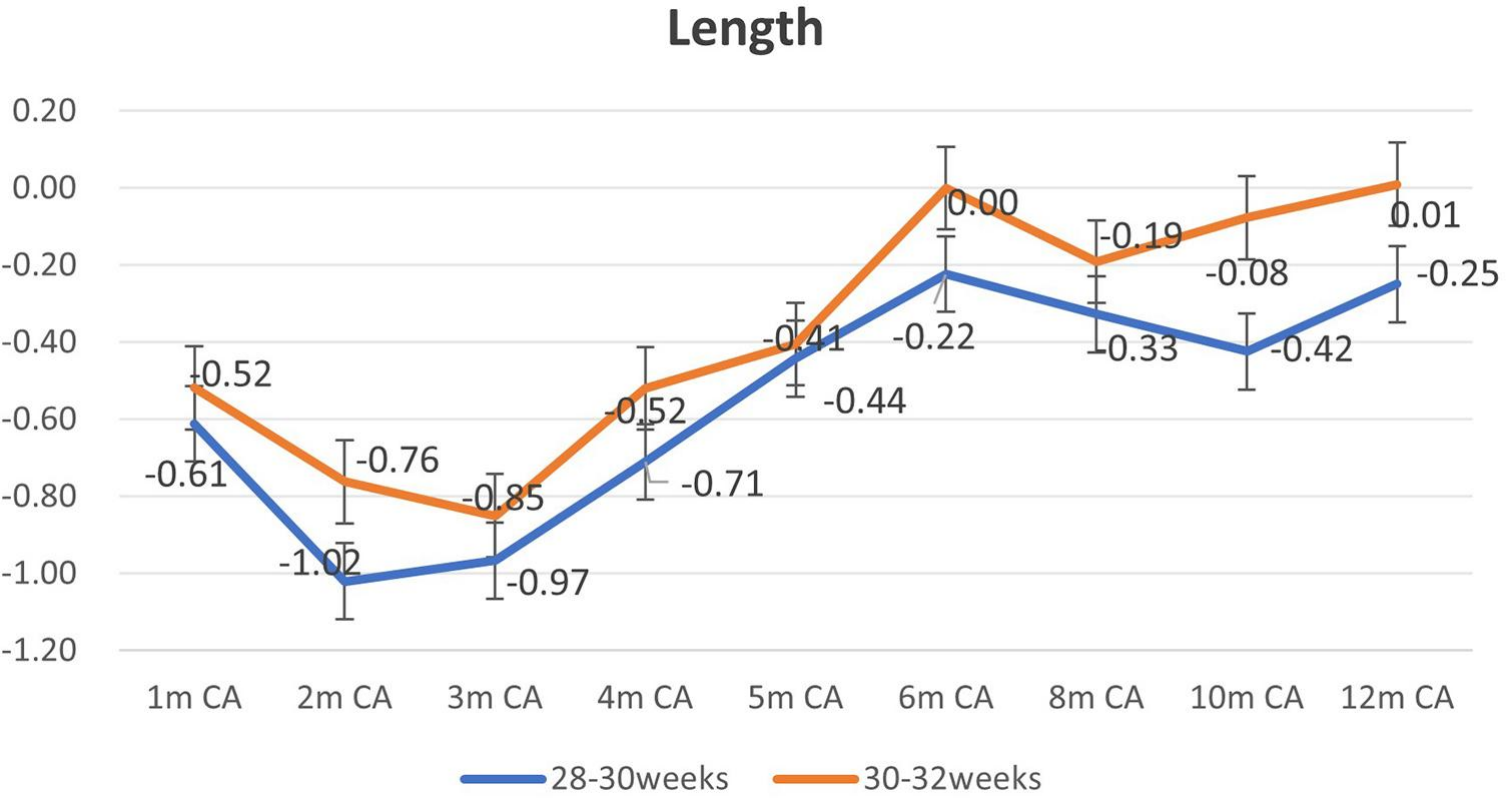
Comparison of Z-scores between gestational age and length.

**Figure 5:**
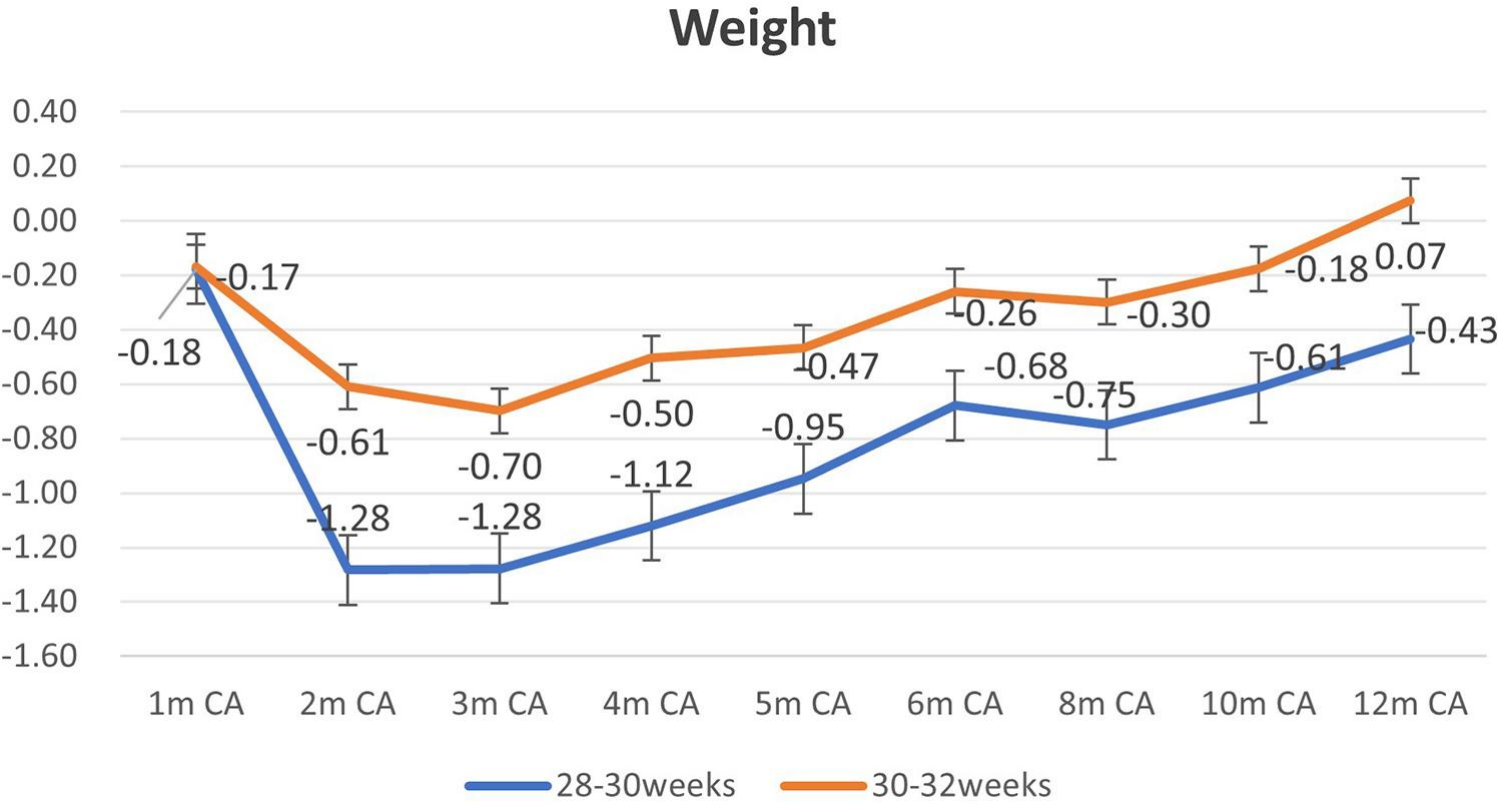
Comparison of Z-scores between gestational age and weight.

**Figure 6:**
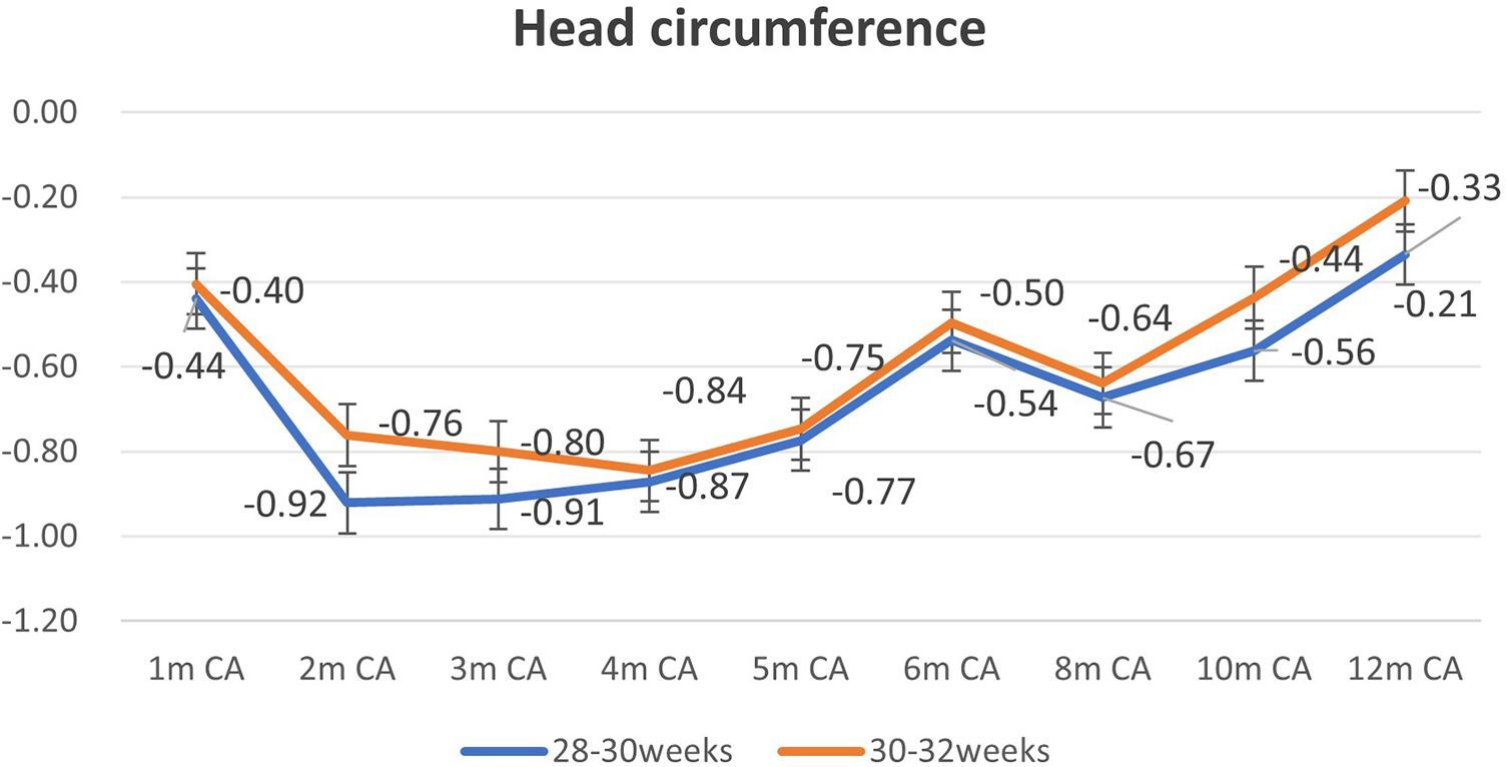
Comparison of Z-scores between gestational age and head circumference.

**Figure 7:**
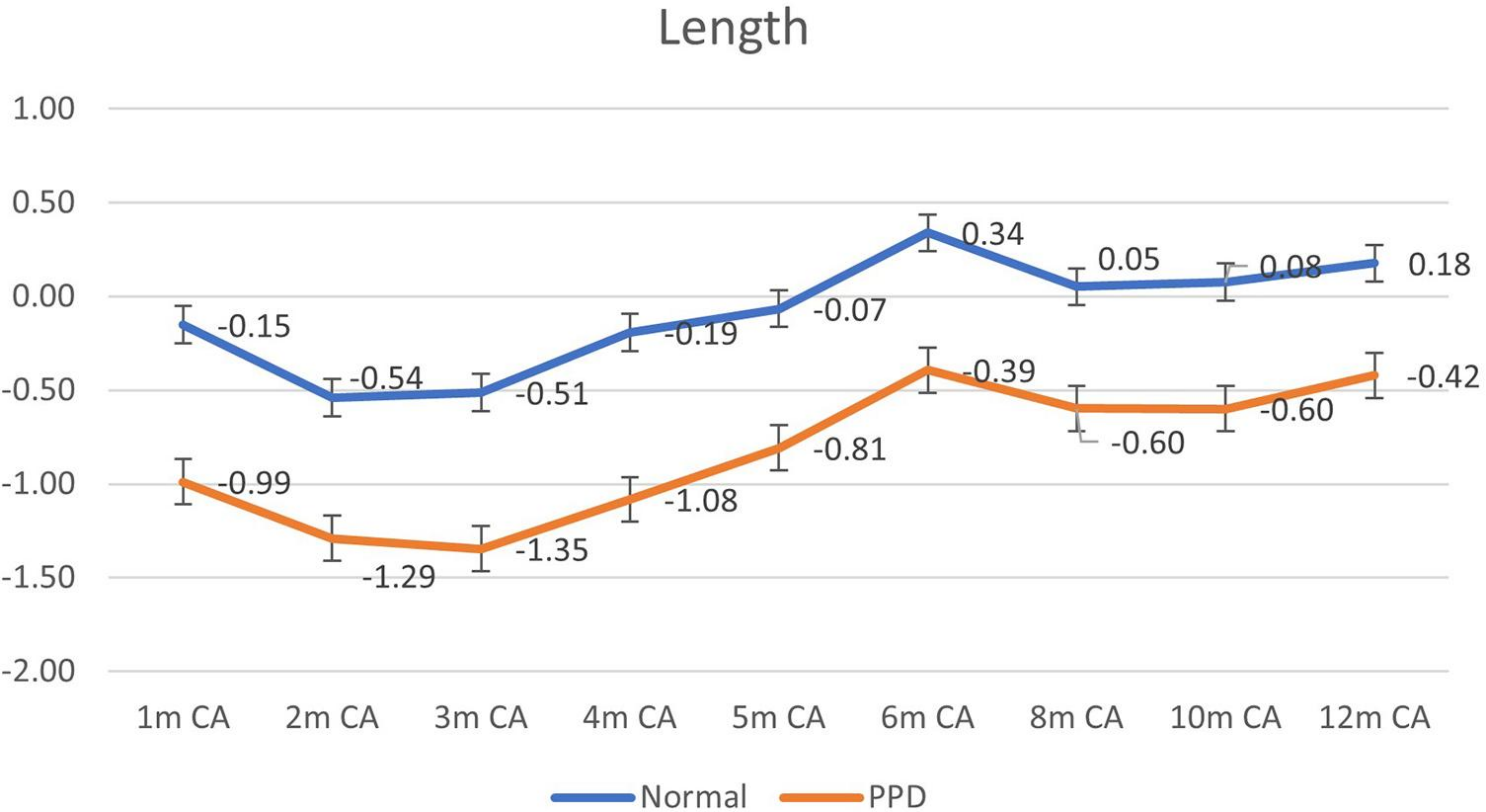
Comparison of Z-scores between maternal PPD and length.

**Figure 8:**
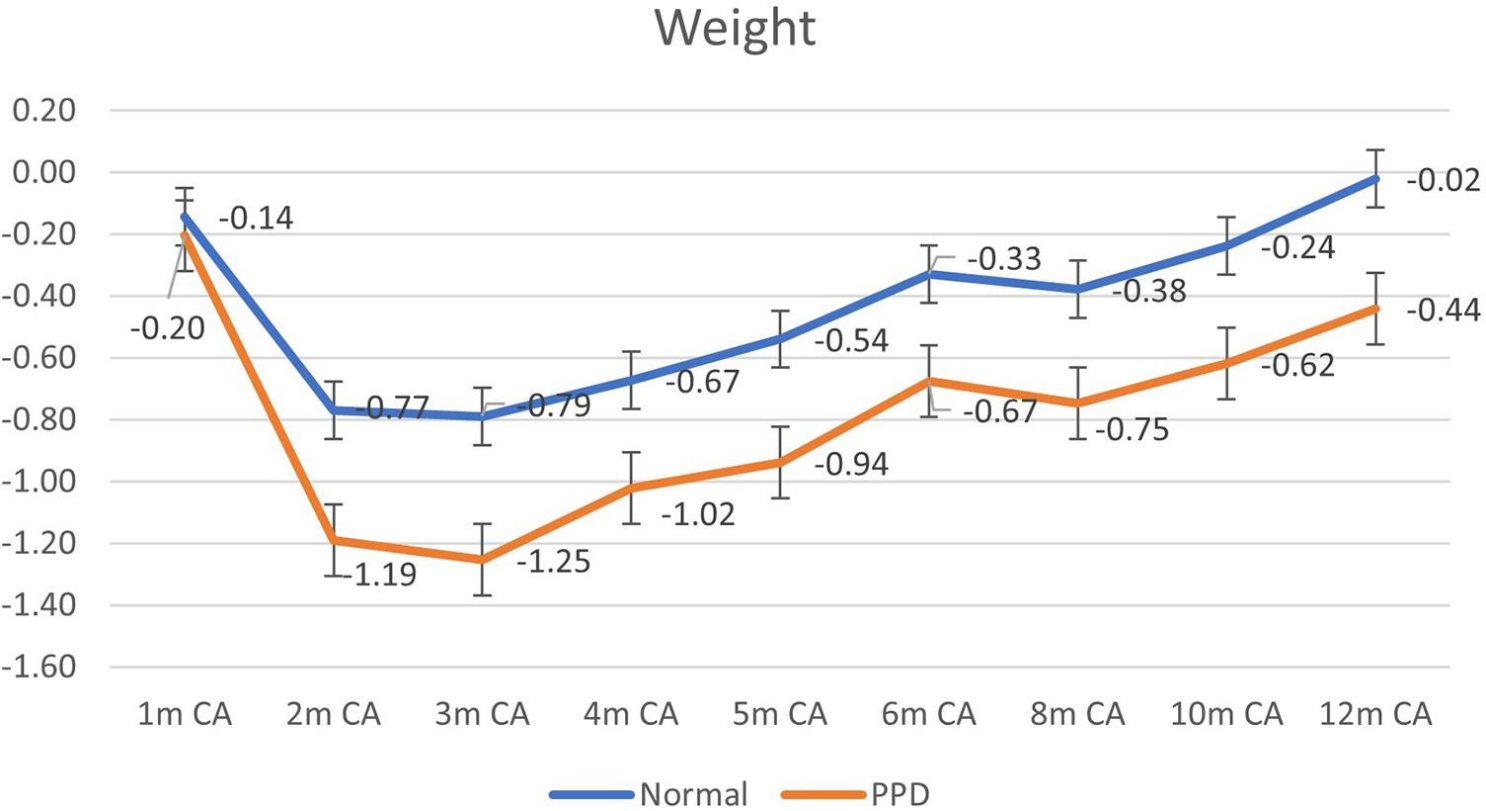
Comparison of Z-scores between maternal PPD and weight.

**Figure 9:**
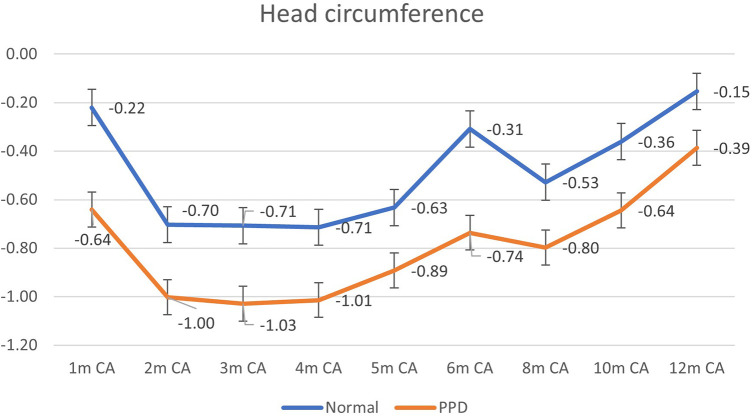
Comparison of Z-scores between maternal PPD and head circumference.

Growth trajectories (weight, length, head circumference z-scores) were analyzed from 1 to 12 months CA stratified by infant sex, gestational age, and maternal PPD status (Figures 1–9). Figures 1 to 3 shows the differences in length, weight, and head circumference between boys and girls at 1–12 months CA. Figures 4 to 6 describe the growth trends of length, weight, and head circumference of very preterm infants aged 1–12 months CA at 28–30 weeks and 30–32 weeks, respectively. Figures 7 to 9 shows the differences in length, weight, and head circumference of very preterm infants at 1–12 months CA with or without postpartum depression.

Multivariate linear regression models of weight, length, and head circumference Z–scores at 1, 3, 6, and 12 months corrected age are presented in Tables 2–5. Maternal PPD was inversely associated with VPI growth at 1, 3, 6, and 12 months CA; higher EPDS scores correlated with smaller head circumference, shorter length, and lower weight. Maternal GDM, subclinical hypothyroidism, and preeclampsia were associated with slower linear growth and head circumference gain. Male sex was consistently linked to poorer growth outcomes compared with female sex. Higher birth weight predicted better growth at 1 month CA. The 1-minute Apgar score, multiple birth status, and exclusive breastfeeding were associated with improved linear growth. CHD was associated with lower weight at 1 month CA and impaired weight gain.

**Table 2:** Multiple regression analysis of physical growth at 1 month CA (*n* = 223).

*Predictors*	*Outcomes: 1 month CA*					
	*Weight*		*Length*		*Head circumference*	
	B ± SE	*p*	B ± SE	*p*	B ± SE	*p*
Maternal factors						
Age	0.03 ± 0.04	*0*.*410*	0.01 ± 0.02	*0*.*802*	0.02 ± 0.03	*0*.*408*
PPD	−0.06 ± 0.04	***0***.***016***	−0.05 ± 0.02	***0***.***002***	−0.07 ± 0.03	***0***.***008***
GDM	0.06 ± 0.35	*0*.*986*	−0.05 ± 0.14	*0*.*714*	−0.17 ± 0.25	*0*.*492*
preeclampsia	−0.45 ± 0.43	*0*.*171*	−0.92 ± 0.87	*0*.*294*	0.34 ± 0.61	*0*.*283*
Subclinical hypothyroidism	−0.61 ± 0.72	*0*.*394*	2.05 ± 0.90	***0***.***025***	0.32 ± 1.02	*0*.*756*
Infant factors						
GA (weeks)	*−0.17* *±* *0.18*	*0*.*344*	*0.22* *±* *0.07*	***0***.***003***	*0.03* ± *0.13*	*0*.*814*
Sex (codes)	*−0.99* *±* *0.34*	***0***.***004***	*−0.07* *±* *0.14*	***0***.***029***	*−0.25* *±* *0.24*	***0***.***032***
Birth weight (gram)	*0.02* *±* *0.01*	***0***.***023***	*0.01* *±* *0.00*	***0***.***024***	*0.01* ± *0.01*	*0*.*070*
1 min Apgar score	*0.10* *±* *0.10*	*0*.*919*	*0.03* *±* *0.04*	*0*.*427*	*0.05* ± *0.07*	*0*.*944*
Twins	*−0.01* *±* *0.33*	*0*.*998*	*0.06* *±* *0.14*	*0*.*640*	*0.11* ± *0.24*	*0*.*635*
Congenital heart disease	*0.04* *±* *0.33*	*0*.*912*	*0.02* *±* *0.14*	*0*.*900*	*−0.27* *±* *0.24*	*0*.*258*
Feeding patterns during hospitalization	*−0.41* *±* *0.26*	*0*.*873*	*−0.18* *±* *0.11*	***0***.***004***	*0.21* ± *0.19*	*0*.*251*

GA, gestational age; PPD, postnatal depression; GDM, gestational diabetes mellitus; *β*±SE, unstandardised regression coefficient ± standard error. Associations at *P* < 0.05 are indicated in bold.

**Table 3:** Multiple regression analysis of physical growth at 3 months CA (*n* = 223).

*Predictors*	*Outcomes: 3 months CA*					
	*Weight*		*Length*		*Head circumference*	
	B ± SE	*p*	B ± SE	*p*	B ± SE	*p*
Maternal factors						
Age	0.02 ± 0.02	*0*.*414*	0.02 ± 0.02	*0*.*879*	−0.02 ± 0.03	*0*.*430*
PPD	−0.01 ± 0.02	*0*.*509*	−0.06 ± 0.02	***0***.***002***	−0.04 ± 0.02	***0***.***038***
GDM	0.07 ± 0.17	*0*.*655*	0.09 ± 0.18	*0*.*612*	0.38 ± 0.22	*0*.*090*
preeclampsia	−0.05 ± 0.39	*0*.*662*	−0.53 ± 0.69	*0*.*069*	−0.77 ± 0.52	*0*.*262*
Subclinical hypothyroidism	−0.16 ± 0.84	*0*.*849*	−3.31 ± 1.11	***0***.***003***	1.38 ± 1.14	*0*.*228*
Infant factors						
GA (weeks)	*−0.16* *±* *0.08*	*0*.*053*	*0.12* *±* *0.09*	*0*.*163*	*−0.63* *±* *0.21*	***0***.***003***
Sex (codes)	*−0.82* *±* *0.16*	***<0***.***001***	*−0.54* *±* *0.17*	***0***.***002***	*−0.54* *±* *0.19*	***0***.***005***
Birth weight (gram)	*0.00* *±* *0.00*	***0***.***001***	*0.00* *±* *0.00*	***0***.***013***	*0.00* *±* *0.00*	***<0***.***001***
1 min Apgar score	*−0.04* *±* *0.05*	*0*.*359*	*0.04* *±* *0.05*	***0***.***014***	*−0.11* *±* *0.06*	*0*.*094*
Twins	*−0.01* *±* *0.16*	*0*.*973*	*−0.36* *±* *0.18*	***0***.***039***	*−0.20* *±* *0.21*	*0*.*357*
Congenital heart disease	*−0.18* *±* *0.16*	*0*.*259*	*−0.08* *±* *0.18*	*0*.*642*	*−0.42* *±* *0.21*	***0***.***045***
Feeding patterns during hospitalization	*0.11* *±* *0.12*	*0*.*362*	*−0.24* *±* *0.14*	***0***.***011***	*0.05* *±* *0.16*	*0*.*766*

GA, gestational age; PPD, postnatal depression; GDM, gestational diabetes mellitus; *β*±SE, unstandardised regression coefficient ± standard error. Associations at *P* < 0.05 are indicated in bold.

**Table 4:** Multiple regression analysis of physical growth at 6 months CA (*n* = 223).

*Predictors*	*Outcomes: 6 months CA*					
	*Weight*		*Length*		*Head circumference*	
	B ± SE	*p*	B ± SE	*p*	B ± SE	*p*
Maternal factors						
Age	−0.02 ± 0.02	*0*.*153*	0.03 ± 0.02	*0*.*098*	0.02 ± 0.02	*0*.*402*
PPD	−0.11 ± 0.02	***0***.***005***	−0.05 ± 0.02	***0***.***008***	−0.03 ± 0.02	***0***.***041***
GDM	−0.12 ± 0.15	*0*.*404*	0.09 ± 0.17	*0*.*599*	0.14 ± 0.17	*0*.*417*
preeclampsia	−0.33 ± 0.39	*0*.*498*	−0.63 ± 0.61	*0*.*299*	−0.78 ± 0.64	*0*.*227*
Subclinical hypothyroidism	−0.17 ± 0.84	*0*.*746*	2.32 ± 1.00	*0*.*023*	1.81 ± 1.06	*0*.*091*
Infant factors						
GA (weeks)	*−0.11* *±* *0.07*	*0*.*145*	*0.07* ± *0.09*	*0*.*408*	*−0.09* *±* *0.08*	*0*.*310*
Sex (codes)	*−0.62* *±* *0.14*	***<0***.***001***	*−0.62* *±* *0.17*	***<0***.***001***	*−0.49* *±* *0.17*	***0***.***003***
Birth weight (gram)	*0.00* *±* *0.00*	***0***.***005***	*0.00* ± *0.00*	***0***.***002***	*0.00* *±* *0.00*	***0***.***004***
1 min Apgar score	*0.05* *±* *0.04*	*0*.*210*	*0.03* ± *0.05*	***0***.***001***	*−0.02* *±* *0.05*	*0*.*751*
Twins	*0.15* *±* *0.14*	*0*.*298*	*0.37* ± *0.17*	***0***.***026***	*0.08* *±* *0.16*	*0*.*622*
Congenital heart disease	*−0.13* *±* *0.14*	*0*.*343*	*−0.07* *±* *0.16*	*0*.*637*	*−0.14* *±* *0.16*	*0*.*405*
Feeding patterns during hospitalization	*0.09* *±* *0.11*	*0*.*428*	*0.14* ± *0.13*	***0***.***025***	*0.21* *±* *0.13*	*0*.*091*

GA, gestational age; PPD, postnatal depression; GDM, gestational diabetes mellitus; *β*±SE, unstandardised regression coefficient ± standard error. Associations at *P* < 0.05 are indicated in bold.

**Table 5:** Multiple regression analysis of physical growth at 12 months CA (*n* = 223).

*Predictors*	*Outcomes: 12 months CA*					
	*Weight*		*Length*		*Head circumference*	
	B ± SE	*p*	B ± SE	*p*	B ± SE	*p*
Maternal factors						
Age	0.03 ± 0.02	*0*.*110*	0.01 ± 0.02	*0*.*618*	−0.03 ± 0.02	*0*.*065*
PPD	−0.02 ± 0.02	***0***.***025***	−0.16 ± 0.04	***0***.***016***	−0.03 ± 0.04	*0*.*435*
GDM	−0.12 ± 0.15	*0*.*405*	−0.02 ± 0.18	*0*.*929*	0.21 ± 0.15	*0*.*145*
preeclampsia	−0.46 ± 0.40	*0*.*164*	−0.28 ± 0.08	***0***.***007***	0.02 ± 0.56	*0*.*970*
Subclinical hypothyroidism	−0.57 ± 0.65	*0*.*479*	−1.65 ± 0.87	*0*.*060*	−2.58 ± 0.93	***0***.***006***
Infant factors						
GA (weeks)	*−0.18* *±* *0.07*	***0***.***009***	*0.07* *±* *0.09*	*0*.*406*	*−0.05* *±* *0.07*	*0*.*441*
Sex (codes)	*−0.58* *±* *0.14*	***<0***.***001***	*−0.79* *±* *0.17*	***<0***.***001***	*−0.37* *±* *0.14*	***0***.***008***
Birth weight (gram)	*0.00* *±* *0.00*	***0***.***001***	*0.00* *±* *0.00*	*0*.*001*	*0.00* *±* *0.00*	***<0***.***001***
1 min Apgar score	*−0.04* *±* *0.04*	*0*.*313*	*0.02* *±* *0.05*	***0***.***037***	*0.01* *±* *0.04*	*0*.*848*
Twins	*0.06* *±* *0.14*	*0*.*679*	*0.23* *±* *0.17*	***0***.***008***	*0.09* *±* *0.14*	*0*.*493*
Congenital heart disease	*−0.23* *±* *0.14*	*0*.*090*	*−0.22* *±* *0.17*	*0*.*192*	*−0.06* *±* *0.14*	*0*.*628*
Feeding patterns during hospitalization	*−0.07* *±* *0.11*	*0*.*507*	*−0.36* *±* *0.13*	***0***.***007***	*0.31* *±* *0.11*	***0005***

GA, gestational age; PPD, postnatal depression; GDM, gestational diabetes mellitus; *β*±SE, unstandardised regression coefficient ± standard error. Associations at *P* < 0.05 are indicated in bold.

## 6 Multivariable regression analyses

Multiple linear regression revealed that preeclampsia and low birth weight predicted reduced weight gain; low birth weight and CHD were associated with attenuated linear growth; and maternal PPD correlated with slower head circumference growth (Table 6).

**Table 6:** Factors influencing 0–12 months CA growth parameters (*n* = 223).

*Predictors*	*Outcomes: 0–12 months CA delta z-scores*					
	*Weight*		*Length*		*Head circumference*	
	B ± SE	*p*	B ± SE	*p*	B ± SE	*p*
Maternal factors						
Age	−0.01 ± 0.04	*0*.*759*	0.09 ± 0.02	*0*.*683*	−0.03 ± 0.03	*0*.*257*
PPD	−0.05 ± 0.04	*0*.*185*	−0.03 ± 0.02	*0*.*889*	0.04 ± 0.02	***<0***.***001***
GDM	−0.21 ± 0.37	*0*.*573*	−0.05 ± 0.19	*0*.*812*	0.27 ± 0.24	*0*.*261*
preeclampsia	0.71 ± 0.27	***0***.***009***	0.10 ± 0.58	*0*.*862*	0.21 ± 0.49	*0*.*306*
Subclinical hypothyroidism	0.51 ± 0.60	*0*.*395*	−0.40 ± 0.96	*0*.*675*	−2.17 ± 1.07	*0*.*045*
Infant factors						
GA (weeks)	*0.05* *±* *0.18*	*0*.*797*	*−0.04* *±* *0.10*	*0*.*663*	*−0.09* *±* *0.12*	*0*.*439*
Sex (codes)	*0.53* *±* *0.35*	*0*.*133*	*−0.53* *±* *0.19*	***0***.***005***	*−0.12* *±* *0.23*	*0*.*591*
Birth weight (gram)	*−0.01* *±* *0.01*	***0***.***005***	*0.00* *±* *0.00*	*0*.*176*	*0.00* *±* *0.00*	*0*.*579*
1 min Apgar score	*−0.08* *±* *0.10*	*0*.*451*	*−0.12* *±* *0.05*	***0***.***024***	*−0.03* *±* *0.07*	*0*.*710*
Twins	*0.08* *±* *0.35*	*0*.*814*	*0.26* *±* *0.19*	*0*.*163*	*0.07* *±* *0.23*	*0*.*768*
Congenital heart disease	*−0.38* *±* *0.34*	*0*.*266*	*−0.30* *±* *0.18*	***0***.***030***	*0.01* *±* *0.23*	*0*.*965*
Feeding patterns during hospitalization	*−0.03* *±* *0.26*	*0*.*899*	*0.10* *±* *0.14*	*0*.*481*	*0.01* *±* *0.18*	*0*.*942*

GA, gestational age; PPD, postnatal depression; GDM, gestational diabetes mellitus; *β*±SE, unstandardised regression coefficient ± standard error. Associations at *P* < 0.05 are indicated in bold.

Logistic regression of growth patterns (1–12 months CA delta z-scores) showed that maternal PPD predicted impaired head circumference growth, gestational age predicted length and head circumference growth, and birth weight was inversely associated with weight gain (Table 7).

**Table 7:** Factors influencing 0–12 growth parameters (*n* = 223).

*Predictors*	*Outcomes: 0–12 months weight, length, head circumference gain patterns*					
	*weight*		*length*		*head circumference*	
	*OR (95%CI)*	*p*	*OR (95%CI)*	*p*	*OR (95%CI)*	*p*
Maternal factors						
Age	0.89∼1.10	0.887	0.91∼1.07	0.710	0.95∼1.13	0.471
PPD	0.89∼1.01	0.976	0.90∼1.05	0.448	0.58∼0.87	**0**.**010**
GDM	0.13∼1.58	0.210	0.19∼1.84	0.947	0.26∼1.17	0.121
preeclampsia	0.14∼2.12	0.172	0.51∼2.06	0.769	0.53∼2.96	0.602
Subclinical hypothyroidism	0.34∼2.11	0.718	0.54∼2.36	0.746	0.47∼1.99	0.850
Infant factors						
GA (weeks)	0.90∼2.15	0.143	0.42∼0.87	**0**.**007**	0.36∼0.91	**0**.**048**
Sex (codes)	0.34∼2.01	0.670	0.26∼1.03	0.059	0.50∼2.22	0.884
Birth weight (gram)	*1.001∼1.005*	***0***.***007***	*0.998∼1.001*	*0*.*447*	*0.99∼1.002*	*0*.*749*
1 min Apgar score	*0.92∼1.48*	*0*.*209*	*0.90∼1.36*	*0*.*329*	*0.73∼1.18*	*0*.*549*
Twins	*0.62∼3.41*	*0*.*390*	*0.69∼2.66*	*0*.*373*	*0.44∼1.87*	*0*.*785*
Congenital heart disease	*0.35∼1.95*	*0*.*660*	*0.35∼1.34*	*0*.*276*	*0.68∼2.98*	*0*.*345*
Feeding patterns during hospitalization	*0.26∼7.66*	*0*.*445*	*0.58∼6.50*	*0*.*284*	*0.31∼6.62*	*0*.*648*

GA, gestational age; PPD, postnatal depression; GDM, gestational diabetes mellitus; OR (95%CI), Lower limit∼upper limit. Associations at *P* < 0.05 are indicated in bold.

### 6.1 Factors influencing birth parameters

Maternal postnatal depression, clinical hypothyroidism, gestational diabetes mellitus (GDM), preeclampsia, and congenital heart disease in very preterm infants (VPIs) were identified as independent risk factors for impaired physical growth. In contrast, female sex, higher gestational age, greater birth weight, elevated 1-minute Apgar score, and exclusive breastfeeding were protective factors associated with improved anthropometric growth outcomes.

## 7 Discussion

This prospective cohort study demonstrates that growth trajectories of weight, length, and head circumference in VPI from 1 to 12 months CA follow a decelerating curve. Male sex, maternal PPD, and lower gestational age (28–30 weeks) were associated with poorer growth. Multivariable models confirmed that maternal PPD, GDM, preeclampsia, subclinical hypothyroidism, male sex, multiple birth, low birth weight, younger gestational age, and CHD were independent risk factors for growth restriction. In contrast, higher 1-minute Apgar scores and exclusive breastfeeding in the NICU were protective factors. Among all variables, maternal PPD, gestational age, and birth weight had the strongest effects on 12-month growth outcomes.

## 8 Maternal factors associated with VPI growth

Maternal postnatal depression is highly prevalent among mothers of very preterm infants and may exert adverse effects on maternal quality of life and impair mother–infant interactions. Previous studies have demonstrated that traumatic responses at 6 months and clinically significant anxiety and depressive symptoms at 6 and 12 months postpartum are associated with the quality of mother–infant interaction at 12 months of age. Maternal postpartum depression is linked to decreased breast milk production (11), reduced maternal responsiveness to infant cues, and in severe cases, harmful caregiving behaviors. One study indicated that maternal postnatal depression impairs responsive feeding, which requires accurate recognition and timely response to infant hunger and satiety signals (12). Furthermore, maternal depression is frequently associated with shorter breastfeeding duration due to diminished self-confidence and reduced self-efficacy. Notably, VPI are particularly vulnerable and demanding to care for; accordingly, the prevalence of postpartum depression is significantly higher among mothers of VPI than among those of term infants (13). The present study confirmed that maternal postnatal depression is negatively associated with physical growth in VPI, which is consistent with prior evidence.

Other maternal factors including cervical insufficiency and placental abruption showed no statistically significant associations with postnatal growth in this study. These conditions are mainly acute, intrapartum events leading to preterm delivery rather than sustained metabolic or hormonal disturbances; thus, their impact on long-term postnatal growth appears limited. In the present study, maternal comorbidities during pregnancy, including gestational diabetes mellitus (GDM), preeclampsia, and subclinical hypothyroidism, were independently associated with impaired somatic growth in VPI. In a longitudinal study of 438 infants born to mothers with GDM, male infants exhibited higher body weight and body mass index than female infants (14). Infants exposed to maternal GDM displayed higher BMI during the first 2 years of life relative to the WHO 2006 growth standards (15). Because the present study focused on VPI rather than term infants, the observed growth patterns differed. Our results showed that maternal GDM did not significantly affect weight or length gain in VPI. However, maternal GDM was positively associated with head circumference growth in VPI at 1 month corrected age (CA), resulting in larger head circumference in exposed infants.

Mothers with preeclampsia often present with advanced maternal age, obesity, and/or underlying vascular disorders (16). Previous research has shown that maternal hypertension during pregnancy increases the risk of placental vasculopathy (17) and predisposes infants to a range of postnatal complications, including respiratory distress, necrotizing enterocolitis, sepsis, and even death (18). These complications prolong the clinical course of VPI, complicate postnatal care, and ultimately hinder growth and development (19).

Maternal hypothyroidism reduces circulating levels of triiodothyronine (T3) and thyroxine (T4) (20), thereby decreasing transplacental transfer of thyroid hormones to the fetus. Fetal thyroid hormone deficiency impairs the metabolism of key nutrients including glucose, lipids, and proteins (21), increasing the risk of low birth weight, shorter body length, and smaller head circumference. Postnatally, affected infants require a longer period to achieve catch-up growth. In addition, thyroid hormones are essential for normal bone growth and mineralization (22). Consequently, infants born to mothers with hypothyroidism exhibit lower thyroid hormone levels compared with those born to euthyroid mothers, which further compromises linear growth and weight gain.

## 9 Infant factors affecting physical growth in VPI

The present study identified several infant-related determinants of physical growth in VPI, including birth weight, gestational age at birth, sex, 1-minute Apgar score, multiple birth status, congenital heart disease (CHD), and in-hospital feeding practices. We observed a positive correlation between birth weight and length and weight z-scores at 12 months CA. Multiple linear regression revealed that length and weight standard deviation scores (SDS) at 12 months CA were negatively associated with birth weight below the 10th percentile. These findings suggest that early childhood catch-up growth is more strongly associated with reduced length at birth than with weight or gestational age at birth (23). Small for gestational age (SGA) is conventionally defined as birth weight below the 3rd or 10th percentile for gestational age. Most studies have used birth weight percentile to evaluate catch-up growth in SGA infants (24); however, our results indicate that birth weight itself may be a more critical indicator for assessing linear and weight growth. Accordingly, birth weight should be incorporated into growth definition and longitudinal follow-up protocols for VPI.

Previous studies have documented substantial changes in head circumference, length, and weight gain during the first year of life in preterm infants, depending on whether they undergo catch-up or catch-down growth. One study reported that term SGA infants may achieve catch-up growth as early as 2 months of age (25). However, lower gestational age at birth is associated with a longer time required to complete catch-up growth (26), and some VPI fail to achieve full catch-up growth by 12 months CA. Early growth trajectories are predictive of long-term developmental outcomes and should not be overlooked. Early nutritional support, adequate neonatal weight gain, and prevention of the early catabolic state have been linked to improved long-term outcomes in VPI (14). Our findings indicate that the degree of growth restriction correlates with the time required to achieve expected growth for gestational age. For smaller VPI, intensified early nutritional support is warranted (27) to promote weight gain, facilitate earlier catch-up growth, prevent growth faltering at 12 months CA, and reduce the risk of subsequent neurodevelopmental delay.

Growing evidence indicates sex-specific hormonal responses to intrauterine and postnatal stress. The male fetus grows more rapidly and is more sensitive to hormonal regulation from early gestation. In contrast, female fetuses tend to maintain tighter homeostatic control to improve survival. Although both *in utero* growth and sex influence postnatal growth in VPI, the underlying mechanisms remain incompletely understood (28). The present study showed that male VPI had significantly lower z-scores for head circumference, length, and weight than females, consistent with findings by Chou et al. (29) It has also been proposed that the poorer growth outcomes in male VPI may be partly attributable to insufficient nutritional intake after discharge (30). Therefore, sex-specific nutritional strategies should be considered for VPI to ensure adequate intake and prevent postnatal growth restriction.

The 1-minute Apgar score reflects the immediate neonatal condition and early complication risk. Approximately 70% of VPI have a 1-minute Apgar score < 7 (31), which increases susceptibility to hypoxic–ischemic encephalopathy, hypoglycemia, and aspiration pneumonia. These conditions prolong clinical management and hinder growth. A lower 1-minute Apgar score is associated with higher mortality in preterm infants and similarly predicts poorer physical growth. Therefore, VPI with low 1-minute Apgar scores require early nutritional intervention in addition to respiratory support to optimize growth outcomes.

A national-level study of 9314 VPI compared perinatal characteristics and outcomes between twin and singleton infants. Twins had a higher prevalence of adverse neonatal outcomes, including mortality, respiratory distress syndrome (RDS), surfactant administration, and longer NICU stays. Twins also had lower median gestational age and lower mean birth weight than singletons (32). Consequently, twins require a longer period to achieve catch-up growth. Another cohort study reported that multiple preterm infants were more likely to receive mixed feeding than singletons (33). Because breast milk intake is positively associated with physical growth, higher breast milk proportion predicts better growth. However, breast milk supply from mothers of twins is often insufficient to meet the needs of both infants (34), which partially explains the higher rate of growth faltering in preterm twins relative to singletons.

Congenital heart disease (CHD) refers to structural anomalies of the heart or great vessels present at birth, affecting approximately 1% of live births in developed countries. A study of 225 children with CHD documented widespread delays in weight gain and linear growth (35). Feeding and swallowing difficulties are common comorbidities in infants with CHD, who require more time to develop coordinated sucking, swallowing, and breathing patterns than healthy term infants (36); feeding also increases oxygen consumption and cardiac workload, limiting intake and slowing linear growth. Chronic hypoxemia, insufficient systemic perfusion, higher energy expenditure, and recurrent respiratory infections further compromise nutrient utilization and bone growth (34). In the present study, CHD did not significantly affect single-time-point growth but was significantly associated with poorer linear growth velocity (0–12 months CA delta z-scores). These findings confirm that growth impairment is prevalent among infants with CHD and support the integration of feeding and growth assessment into routine clinical care.

Breastfeeding is strongly recommended worldwide owing to its unique benefits for preterm infant health (37). For VPI, breast milk provides not only nutrition but also immunological and bioactive factors that enhance resistance and support development. After birth, VPI admitted to the NICU undergo extensive metabolic and homeostatic adaptation to the extrauterine environment, which may increase long-term risks of metabolic and neurodevelopmental disorders (38). Some studies have found no significant differences in weight, length, or head circumference z-scores between breastfed and formula-fed preterm infants (39). Brownell et al. (40–43) investigated the effects of donor milk, maternal milk, and preterm formula on growth rates and reported that weight z-score change decreased with higher donor milk intake but increased with higher formula proportion. In the present study, exclusive breastfeeding during hospitalization showed no significant effect on weight gain but was positively associated with linear growth and head circumference gain. These findings support the benefits of exclusive breastfeeding for physical growth in VPI and highlight the importance of promoting exclusive breastfeeding in this high-risk population.

## 10 Conclusions

In conclusion, physical growth in VPI is shaped by a complex interplay of maternal and infant factors. Male sex, lower gestational age at birth, lower birth weight, and lower 1-minute Apgar score are independent infant-related risk factors. Maternal subclinical hypothyroidism, gestational diabetes mellitus, preeclampsia, and especially postpartum depression strongly predict impaired growth in VPI. In contrast, factors causing acute preterm delivery such as cervical insufficiency and placental abruption show no significant impact on long-term postnatal growth, likely because they represent transient intrapartum events rather than sustained metabolic or endocrine disturbances. These findings underscore the need for integrated interventions targeting maternal mental health, obstetric complication management, and individualized nutritional support to optimize growth and developmental outcomes in VPI.

## Data Availability

The original contributions presented in the study are included in the article/Supplementary Material, further inquiries can be directed to the corresponding author.
