# Supplementary material for: Determinants of growth parameters at 12-month corrected age among very preterm infants in China: a retrospective cohort study
Source: Front Pediatr. 2026 Jul 6;14:1847139. doi: 10.3389/fped.2026.1847139 (PMC13381610; doi:10.3389/fped.2026.1847139)
Supplement: Supplementary file 1 [file Table1.docx]

**Appendix 1**

**General information questionnaire for preterm infants**

**Mother**

Age:_________ Delivery gestational age:_________

Education background:____________________

Delivery mode:_________ G______ P_____

Pregnancy complications:_________

□ Cervical dysfunction □Preeclampsia

□Gestational diabetes mellitus (GDM) □Subclinical hypothyroidism

□Pregnancy-induced hypertension syndrome □PROM

□Other complications_________

**Father**

Age:_________ Education background:____________________

**Infant**

Gestational age:____________ Birth weight:__________

Gender:________ Apgar scores at 1 min:______ 5 min______

Twins:□No □Yes Length of stay:_____________days

Congenital heart disease:□No □Yes __________________

Feeding patterns during hospitalization: □Exclusive breastfeeding

□Mixed feeding

□Formula feeding

Neonatal complications:

□Acute respiratory distress syndrome (NRDS)

□Hypoglycemia

□Hyperbilirubinemia

□BPD

□NEC

□anemia
